# Initial Progression-Free Survival after Non-First Line TKIs Therapy Potentially Guides Immediate Treatment after Its Failure in Advanced Non-Small Cell Lung Cancer

**DOI:** 10.3969/j.issn.2095-3941.2012.01.007

**Published:** 2012-03

**Authors:** Fang Wang, Gui-fang Guo, Hui-juan Qiu, Wen-zhuo He, Fei-fei Zhou, Xu-xian Chen, Pi-li Hu, Bei Zhang, Chen-xi Yin, Li Zhang, Liang-ping Xia

**Affiliations:** 1 State Key Laboratory of Oncology in South China, Guangzhou 510060, China; 2 VIP Region, Sun Yat-sen University Cancer Center, Guangzhou 510060, China; 3 Tumor Center, The Foshan First People’s Hospital, Foshan 528000, China; 4 Department of Medical Oncology, Sun Yat-sen University Cancer Center, Guangzhou 510060, China

**Keywords:** lung neoplasm, chemotherapy, survival analysis, erlotinib

## Abstract

**Objective:**

The standard therapy after failure of the initial non-first line epidermal growth factor receptor tyrosine kinase inhibitor (EGFR-TKI) treatment in advanced non-small cell lung cancer (NSCLC) has not yet been established. The aim of the current study was to identify whether the 2^nd^ TKI treatment or chemotherapy (paclitaxel-containing or non-paclitaxel regimen) is the appropriate treatment for patients with NSCLC based on the efficacy of the initial TKIs.

**Methods:**

Seventy-two advanced NSCLC patients who had accepted 2^nd^ TKIs or chemotherapy immediately after failure of the initial TKIs in non-first line setting from May 1, 2004 to January 31, 2010 at the Sun Yat-sen University Cancer Center were enrolled. The primary endpoint [2^nd^ progression-free survival (PFS)] and the second endpoint [overall survival (OS)] were compared among the 2^nd^ TKI and chemotherapy groups as well as their subgroups.

**Results:**

(1) Twenty-one patients were treated with 2^nd^ TKIs, and 51 patients were administered chemotherapy after failure of the initial non-first line TKI treatment. There was nonsignificant difference in the responses (*P*=0.900) [2^nd^ PFS (*P*=0.833) and OS (*P*=0.369)] between the 2^nd^ TKI and chemotherapy groups. (2) In the 2^nd^ TKI group, 9 patients exhibited PFS≥7 months. The initial TKI treatment group exhibited a longer 2^nd^ PFS than the other 12 patients with an initial PFS<7 months (7 months *vs.* 2 months, *P*=0.019). However, these groups had nonsignificantly different OS (*P*=0.369). (3) In the chemotherapy group, patients with PFS<5 months exhibited longer 2^nd^ PFS than those with PFS ≥ 5 months in the initial TKI treatment (3 months *vs.* 2 months, *P*=0.039). (4) In the chemotherapy group, patients treated with paclitaxel-containing regimen showed longer 2^nd^ PFS than those treated with non-paclitaxel regimen (5 months *vs.* 2.3 months, *P*=0.043).

**Conclusions:**

Patients with PFS≥7 months or <5 months under the initial TKI treatment potentially benefit from the 2^nd^ TKI treatment or chemotherapy immediately after failure of the non-first line TKIs. The paclitaxel-containing regimen may improve the 2^nd^ PFS. However, more patient samples are urgently needed to validate these findings.

## Introduction

Epidermal growth factor receptor tyrosine kinase inhibitors (EGFR-TKIs) play an important role in the treatment of non-small cell lung cancer (NSCLC), especially in the first line setting where both the objective response rate and progression-free survival (PFS) are better than chemotherapy once the cancer bears the mutant EGFR ^[^^1^^–^^3^^]^. Moreover, the efficacy of EGFR-TKIs was fully demonstrated in non-first line setting regardless of the status of the EGFR mutation ^[^^4^^–^^6^^]^. However, a standard regimen after failure of the non-first line TKI therapy has not been established.

Erlotinib is used initially as a salvage treatment option after the failure of gefitinib. In the first phase II clinical trial in this field conducted by Cho et al. ^[^^7^^]^, the disease control rate (DCR) and response rate (RR) for all patients were 28.6% and 9.5%, respectively. Thus, erlotinib seems to be a potential therapeutic option. However, contradicting results were obtained from another phase II clinical trial with a similar design ^[^^8^^]^, in which the RR was 4.3% and DCR was 8.7%. Hence, erlotinib should not be given routinely after failure of the gefitinib treatment ^[^^9^^]^. However, erlotinib can be an option for a highly selected subset that had benefited from prior gefitinib treatment. This perception is in accordance with those in several other studies ^[^^10^^–^^12^^]^ and is fully confirmed in a pooled analysis of 106 patients. The beneficial population is confined to patients who had shown stable disease (SD) despite gefitinib therapy (*P*=0.0095) and to patients who had PFS of more than 6 months during gefitinib treatment (*P*=0.0261) ^[^^13^^]^. The success of erlotinib may be attributed to its effectiveness in patients who do not respond well to gefitinib, such as those with negative EGFR mutation, squamous cell carcinoma, or a history of smoking^[^^4^^]^. Moreover, erlotinib is used at its maximum tolerated dose, whereas gefitinib is used only at approximately one-third of its maximum tolerated dose in daily practice. The other targeted agents were researched successfully in several studies, such as the cetuximab ^[^^14^^]^ and TKIs combined with cytotoxic agents, such as paclitaxel ^[^^15^^]^. Although chemotherapy is the standard treatment recommended by the National Comprehensive Cancer Network 2011 for NSCLC after failure of TKIs, there are very limited studies in this field. Platinum-based combination or taxane-containing regimen is associated with a higher therapy response, whereas a platinum-based combination linked to a better OS is found in a retrospective study ^[^^16^^]^ for a total of 195 patients with Stage IIIb or IV NSCLC receiving first-line gefitinib. The survival of platinum-based combination regimens exist in patients with mutant EGFR but not with wild-type EGFR. The sensitivity of cisplatin, docetaxel, and pemetrexed were tested in two cell lines with acquired resistance to gefitinib. According to Zhou et al. ^[^^17^^]^, docetaxel or pemetrexed is a more reasonable choice than cisplatin because the overexpression of PI3K, integrin, and DNA restoration gene, as well as the continuous activation of PI3K in this cell line, is found to be correlated with resistance to cisplatin. However, the effectiveness of chemotherapy after failure of the non-first line TKI treatment remains controversial. From the limited clinical data, the alternative strategy between TKIs and cytotoxic chemotherapy is reasonable because the sensitivity of TKIs can be regained following a drug holiday for patients with NSCLC who initially responded to EGFR-TKIs treatment ^[^^18^^]^ and the EGFR mutation status was changed before and after TKIs treatment ^[^^19^^]^.

To date, treatment after initial TKIs in NSCLC is still being challenged, such as who will benefit from the retreatment of TKIs and cytotoxic chemotherapy and what kind of cytotoxic combination will provide better performance. To the best of our knowledge, a study to address such concerns has not been conducted. Thus, we retrospectively compared the efficacy of the retreatment of TKIs (2^nd^ TKIs group) and chemotherapy (chemotherapy group) immediately after failure of the initial non-first line TKI treatment in NSCLC, as well as the efficacy of paclitaxel-containing and non-paclitaxel regimens, to determine the criteria for the selection of appropriate patients that must be treated with 2^nd^ TKIs or chemotherapy.

## Patients and Methods

### Patients

The inclusion criteria for NSCLC patients are as follows: 1) histologically or cytologically proven NSCLC; 2) received the first line standard chemotherapy; 3) failure of the initial non-first line TKI treatment; 4) received TKI therapy or chemotherapy treatment, including paclitaxel-containing or non-paclitaxel regimen from May 1, 2004 to January 31, 2010 at the Sun Yat-sen University Cancer Center; and 5) with adequate hematologic and hepatic/renal functions. Seventy-two patients were enrolled in the current study, including 21 patients who were administered 2^nd^ TKI treatment and 51 patients who received chemotherapy immediately after failure of the initial TKI therapy. Fourteen of the 51 patients had been treated with paclitaxel-containing regimen, whereas the other 37 patients received non-paclitaxel regimen. The basic characteristics of the patients are summarized in **Table 1**.

**Table 1:** The baseline or prognostic characteristics of different groups.

Characteristics		2^nd^ TKIs				Chemotherapy							P*
		Total No.	PFS ≥7	PFS <7	*P*	Total No.	PFS ≥5	PFS <5	*P*	Paclitaxel- containing	Non- paclitaxel	*P*	
Gender					0.528				0.609			0.454	0.010
Male		10	5	5		40	22	18		10	30		
Female		11	4	7		11	7	4		4	7		
Age, years					0.256				0.543			0.078	0.384
<60		10	3	7		30	16	14		11	19		
≥60		11	6	5		21	13	8		3	18		
Surgery history					0.368				0.116			0.663	0.728
Yes		3	2	1		9	3	6		3	6		
No		18	7	11		42	26	16		11	31		
Radiotherapy history					0.056				0.291			0.037	0.121
Yes		12	3	9		19	9	10		2	17		
No		9	6	3		32	20	12		12	20		
Pathological type					0.229				0.193			0.368	0.339
Adenocarcinoma		19	7	12		35	23	12		12	23		
Squamous cell carcinoma		1	1	0		7	4	3		0	7		
S and A^a^		0	0	0		2	0	2		0	2		
BAC^b^		0	0	0		4	1	3		1	3		
NSCLC		1	1	0		3	1	2		1	2		
Initial clinical stage					0.820				0.305			0.227	0.835
I		0	0	0		2	0	2		1	1		
II		2	1	1		5	2	3		1	4		
III		8	4	4		19	12	7		8	11		
IV		11	4	7		25	15	10		4	21		
Smoking history					0.697				0.443			0.375	0.252
Yes		8	3	5		27	14	13		6	21		
No		13	6	7		24	15	9		8	16		
The lines of initial TKIs application					0.380				0.337			0.058	0.753
Maintenance after 1^st^ line		3	0	3		7	2	5		0	7		
2^nd^ line		6	3	3		21	12	9		5	16		
3^nd^ line		9	5	4		16	10	6		8	8		
After the 3^nd^ line		3	1	2		7	5	2		1	6		
Response to 2^nd^ TKIs or chemotherapy					0.171				0.589			0.237	0.900
PR		4	1	3		12	7	5		5	7		
SD		7	5	2		15	10	5		5	10		
PD		10	3	7		24	12	12		4	20		

*, comparison between 2^nd^ TKI group and chemotherapy group; ^a^, adenocarcinoma and squamous cell carcinoma; ^b^, bronchioloalveolar carcinoma.

### Treatment

All patients accepted 250 mg/day gefitinib or 150 mg/day erlotinib in the initial TKI treatment until the disease progression. In the following treatment, TKI was administered as previously mentioned, and the cytotoxic agents were administered under the standard dosages and schedules. No systemic chemotherapy was given during gefitinib/erlotinib treatment in any patient, although some received radiotherapy for metastasis after temporary cessation of TKIs, followed by the resumption of TKIs.

### Assessment of the response and adverse events

Tumor response was evaluated using radiologic examinations according to the Response Evaluation Criteria in Solid Tumors ^[^^20^^]^. The primary endpoint was the 2^nd^ PFS, which was calculated from the date using the 2^nd^ TKIs or chemotherapy to the first date of disease progression or the date of death. The second endpoint was the OS (starting from the date of diagnosis to the date of death or the last date of follow-up).

### Statistical analysis

χ^2^-test was used to compare the characteristics between the groups, and Kaplan-Meier method was used for survival analysis. *P*<0.05 was considered statistically significant. All statistical analyses were conducted using the SPSS 16.0 software package.

## Results

### Efficacy in 2^nd^ TKIs and chemotherapy groups

**Figure 1:**
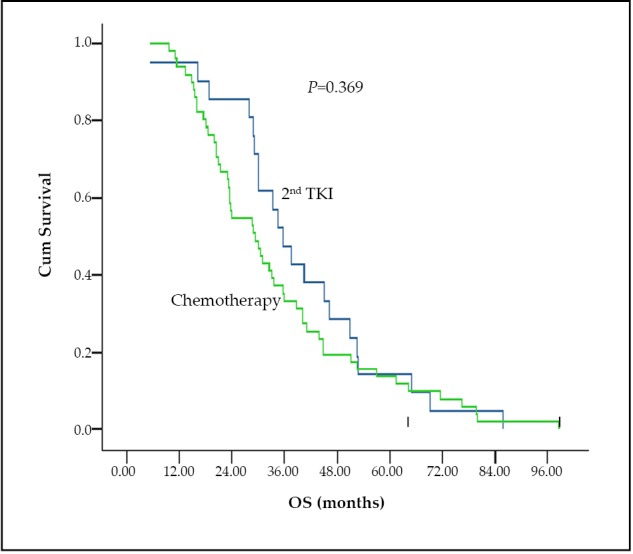
The OS of the 2^nd^ TKI and chemotherapy groups immediately after the failure of initial TKI treatment.

Seventy-two patients were enrolled in the current study, 21 of whom were treated with 2^nd^ TKIs, and the remaining patients accepted chemotherapy immediately after failure of the initial non-first line TKI treatment. All the baseline or prognostic characteristics, including gender, age, surgery history, radiotherapy history, pathological type, staging of disease, smoking history, responses, and so on, were evaluated prior to comparison of results. Most of these characteristics were re-evaluated in the different groups (**Table 1**). All characteristics were balanced, except that more male patients were enrolled in the chemotherapy group (*P*=0.001). There were nonsignificant differences between the 2^nd^ response rates (PR/SD/PD) to the 2^nd^ TKIs or chemotherapy of the two groups. The 2^nd^ PFS and OS were compared in the two groups, which were three months versus three months (*P*=0.833) and 35.7 months versus 25.3 months (*P*=0.369), in the 2^nd^ TKIs and chemotherapy groups, respectively. Neither the difference in the 2^nd^ PFS nor OS between the two groups was significant. The OS values of the two groups are shown in **Figure 1**.

### Efficacy in subgroups of the 2^nd^ TKIs group

**Figure 2:**
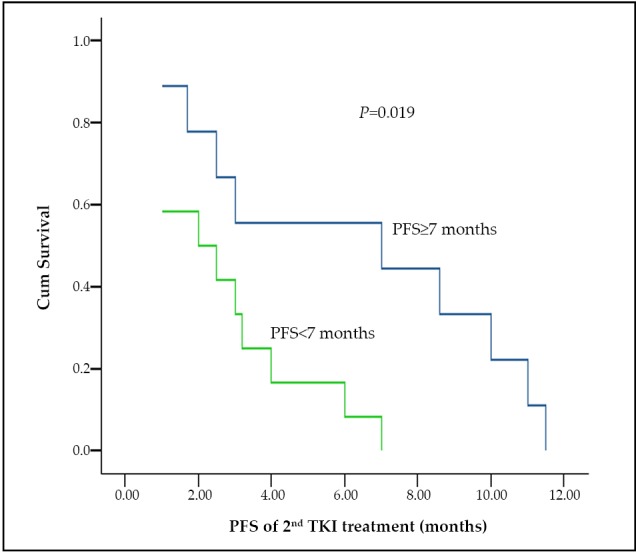
The 2^nd^ PFS of patients with PFS more than or equal to 7 months and less than 7 months after the initial TKI treatment in 2^nd^ TKIs group.

Of the 21 patients in the 2^nd^ TKI group, 17 patients shifted from gefitinib to erlotinib treatment, whereas the remaining patients were given gefitinib continuously after its failure. These patients were classified based on the PFS values under the initial TKIs that are more than or equal to 7 months (9 patients) and less than 7 months (12 patients). The two subgroups have balanced characteristics (**Table 1**). The nonsignificantly different response rates between the two subgroups (*P*=0.171) are listed in **Table 1**. Only the 2^nd^ PFS between the two subgroups were significantly different (7 months *vs.* 2 months, *P*=0.019) (**Figure 2**). The OS between the two groups was nonsignificantly different (35.6 months *vs.* 29.3 months, *P*=0.369).

### Efficacy in subgroups of chemotherapy group

**Figure 3:**
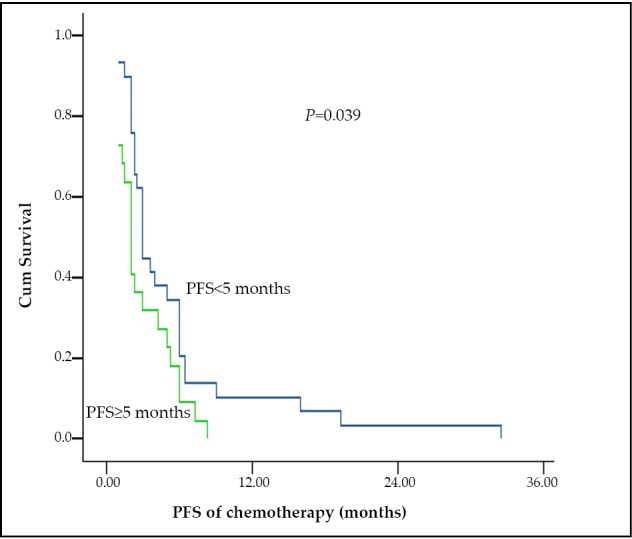
The 2^nd^ PFS of patients with PFS < 5 months and ≥ 5 months in initial TKIs treatment in the chemotherapy group.

**Figure 4:**
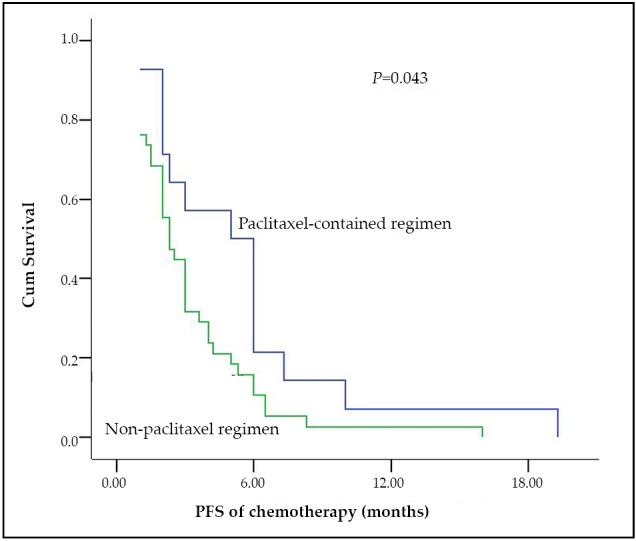
The 2^nd^ PFS of patients with and without paclitaxel-containing regimens in the chemotherapy group.

The 51 patients in the chemotherapy group were classified based on the PFS values after the initial TKIs that are more than or equal to 5 months (29 patients) and less than 5 months (22 patients), and could be classified as patients who accepted paclitaxel-containing regimen chemotherapy (14 patients) or with non-paclitaxel regimen chemotherapy (37 patients). These groups had balanced characteristics, except that more patients were enrolled in the non-paclitaxel group with a history of radiotherapy (*P*=0.037) (**Table 1**). The response rates between the 2 subgroups were nonsignificantly different. Patients with PFS < 5 months showed longer 2^nd^ PFS than patients with PFS ≥ 5 months after the initial TKIs in the chemotherapy group (3 months *vs.* 2 months, *P*=0.039) (**Figure 3**). However, OS between the 2 subgroups was nonsignificantly different (23.5 months *vs.* 33.0 months, *P*=0.465). In the chemotherapy group, patients who received paclitaxel-containing regimen showed a longer 2^nd^ PFS than patients treated with non-paclitaxel regimen and the 2^nd^ PFS were 5 months and 2.3 months (*P*=0.043), respectively (**Figure 4**). The OS between the two subgroups was nonsignificantly different (35.7 months *vs.* 23.6 months, *P*=0.578).

## Discussion

Patients who benefited from the retreatment of TKIs immediately after the failure of non-first line TKIs were identified. SD or PFS of more than 6 months in the initial gefitinib treatment ^[^^7^^, ^^8^^, ^^13^^]^ was the main predictor of patients who will potentially benefit from erlotinib treatment after failure of gefitinib. Patients who failed gefitinib treatment still benefited from continuous gefitinib-containing regimens ^[^^21^^]^. However, the precondition was that patients should get disease control in the initial gefitinib treatment. Considering that most responders failed because of the appearance of new lesions without progression of the pre-existent target lesions ^[^^22^^]^, the continuous gefitinib treatment could control the pre-existent lesions. Riely et al.^[^^23^^]^ found that patients who developed acquired resistance to erlotinib or gefitinib, the stopping TKIs administration resulted in symptomatic progression, increase in standardized uptake value (SUV, max), and increase in tumor size. Interestingly, symptoms improved and SUV (max) decreased after TKIs treatment was restarted. These findings suggest that some tumor cells remain sensitive to EGFR blockade even after acquiring resistance. Beyond these 2 characteristics and using multivariate analysis, Hata et al.^[^^24^^]^ found that the good performance status (PS) and insertion of cytotoxic chemotherapies between gefitinib and erlotinib therapies were significantly predictive factors for longer PFS. Considering that poor PS limited the application of erlotinib ^[^^24^^, ^^25^^]^, such as grade 3/4 adverse events found in 16 (43%) of 38 patients with PS 2/3, dose reduction or interruption was performed in 24 (63%) of 38 poor PS patients, and we considered that the dose intensity was insufficient to maintain the promotion of the benefits of erlotinib. Moreover, poor PS was related to low compliance. Patients who obtained a PFS of more than or equal to 7 months from the initial TKIs treatment will benefit from TKIs retreatment immediately after the failure of the initial TKIs. We infer that longer PFS is a better index than SD or PR to evaluate the initial TKI efficacy because longer PFS means longer efficacy, whereas SD or PR usually reflect short-term efficacy. However, longer time to progression (TTP) does not indicate better survival, as demonstrated in the study conducted by Asami et al. ^[^^26^^]^ Among PR patients, those with TTP<12 months on gefitinib showed significantly longer survival times than those with TTP≥12 months (10.3 months *vs.* 6.4 months; *P*=0.04) from erlotinib salvage treatment. Shifting from gefitinib to erlotinib was common and reasonable because of the superiorities of erlotinib in low dependence population selection ^[^^4^^]^ and maximum tolerated dosage. However, no patients were shifted from erlotinib to gefitinib in the present study.

Patients who benefited from the cytotoxic chemotherapy immediately after the failure of non-first line TKIs were identified. Though the retreatment of TKIs is the focus of studies on the treatment after failure of initial TKIs treatment for NSCLC ^[^^7^^, ^^8^^, ^^13^^, ^^24^^, ^^25^^]^, chemotherapy is the main treatment option once TKIs fails in clinical practice. In the current study, chemotherapy accounted for 70.8% (51/72) and retreatment of TKIs just accounted for 29.2% (21/72). Chemotherapy is the standard option recommended by recent NCCN guidelines for NSCLC patients after failure of the first line TKIs. Although patients with EGFR mutation treated with TKIs in first line achieved longer PFS than chemotherapy, OS in the two groups was similar which could be associated with salvage TK treatment to chemotherapy group and salvage chemotherapy treatment to the TKIs group ^[^^1^^–^^3^^]^. Wu et al. ^[^^16^^]^ demonstrated that platinum-based combination or taxane-containing regimen was associated with a higher therapy response after failure of first line TKIs. However, to the best of our knowledge, there are no studies that have investigated whether chemotherapy is the optimal option after failure of non-first line TKIs. The reaction of chemotherapy is possibly different after failure of the first line TKIs and failure of non-first line TKIs because EGFR mutation testing is obligated in first line TKIs treatment. However, this condition is not required in the non-first line TKI treatment ^[^^1^^, ^^2^^, ^^4^^]^. This phenomenon was the reason why only 2.7% (2/72) of patients with known EGFR mutation status was reported. The lower testing of EGFR mutation was also attributed to the fact that all patients in the current study were subjected to initial TKIs before 2009 when IPASS clinical trial was published ^[^^1^^]^. The present study had demonstrated that patients administered with a paclitaxel-containing regimen had longer 2^nd^ PFS than those who received non-paclitaxel regimen. More patients in the non-paclitaxel group with radiotherapy history (*P*=0.037) might have potentially mild influence on the results because the remaining prognostic factors were balanced. Based on the results from Wu et al. ^[^^16^^]^, the basic research results from Zhou et al. ^[^^17^^]^, and the current results, paclitaxel-containing regimen may obtain longer 2^nd^ PFS immediately after the failure of non-first line TKI treatment.

## Conclusion

Patients with PFS ≥7 months or <5 months after initial TKIs treatment potentially benefit from 2^nd^ TKIs treatment or chemotherapy immediately after the failure of non-first line TKIs. Paclitaxel-containing regimen is a better option. However, studies with more patient samples are urgently needed to validate the findings.

## References

[1] Mok TS Wu YL Thongprasert S Gefitinib or carboplatin-paclitaxel in pulmonary adenocarcinoma. N Engl J Med 2009; 361: 947-957 19692680 10.1056/NEJMoa0810699

[2] Maemondo M Inoue A Kobayashi K Gefitinib or chemotherapy for non-small-cell lung cancer with mutated EGFR. N Engl J Med 2010; 362: 2380-2388 20573926 10.1056/NEJMoa0909530

[3] Zhou C Wu YL Chen G Erlotinib versus chemotherapy as first-line treatment for patients with advanced EGFR mutation-positive non-small-cell lung cancer (OPTIMAL, CTONG-0802): a multicentre, open-label, randomised, phase 3 study. Lancet Oncol 2011; 12: 735-742 21783417 10.1016/S1470-2045(11)70184-X

[4] Shepherd FA Rodrigues Pereira J Ciuleanu T Erlotinib in previously treated non-small-cell lung cancer. N Engl J Med 2005; 353: 123-132 16014882 10.1056/NEJMoa050753

[5] Thatcher N Chang A Parikh P Gefitinib plus best supportive care in previously treated patients with refractory advanced non-small-cell lung cancer: results from a randomised, placebo-controlled, multicentre study (Iressa Survival Evaluation in Lung Cancer). Lancet 2005; 366: 1527-1537 16257339 10.1016/S0140-6736(05)67625-8

[6] Chang GC Tsai CM Chen KC Predictive factors of gefitinib antitumor activity in East Asian advanced non-small cell lung cancer patients. J Thorac Oncol 2006; 1: 520-525 17409911

[7] Cho BC Im CK Park MS Phase II study of erlotinib in advanced non-small-cell lung cancer after failure of gefitinib. J Clin Oncol 2007; 25: 2528-2533 17577030 10.1200/JCO.2006.10.4166

[8] Lee DH Kim SW Suh C Phase II study of erlotinib as a salvage treatment for non-small-cell lung cancer patients after failure of gefitinib treatment. Ann Oncol 2008; 19: 2039-2042 18644828 10.1093/annonc/mdn423 PMC2733114

[9] Sim SH Han SW Oh DY Erlotinib after Gefitinib failure in female never-smoker Asian patients with pulmonary adenocarcinoma. Lung Cancer 2009; 65: 204-207 19110337 10.1016/j.lungcan.2008.11.006

[10] Zhou ZT Xu XH Wei Q Erlotinib in advanced non-small-cell lung cancer after gefitinib failure. Cancer Chemother Pharmacol 2009; 64: 1123-1127 19322567 10.1007/s00280-009-0973-1

[11] Vasile E Tibaldi C Chella A Erlotinib after failure of gefitinib in patients with advanced non-small cell lung cancer previously responding to gefitinib. J Thorac Oncol 2008; 3: 912-914 18670311 10.1097/JTO.0b013e318180275e

[12] Wong AS Soong R Seah SB Evidence for disease control with erlotinib after gefitinib failure in typical gefitinib-sensitive Asian patients with non-small cell lung cancer. J Thorac Oncol 2008; 3: 400-404 18379359 10.1097/JTO.0b013e318168c801

[13] Kaira K Naito T Takahashi T Pooled analysis of the reports of erlotinib after failure of gefitinib for non-small cell lung cancer. Lung Cancer 2010; 68: 99-104 19540616 10.1016/j.lungcan.2009.05.006

[14] Wu JY Yang CH Hsu YC Use of cetuximab after failure of gefitinib in patients with advanced non-small-cell lung cancer. Clin Lung Cancer 2010;11: 257-263 20630828 10.3816/CLC.2010.n.033

[15] Shukuya T Takahashi T Tamiya A Gefitinib plus paclitaxel after failure of gefitinib in non-small cell lung cancer initially responding to gefitinib. Anticancer Res 2009; 29: 2747-2751 19596955

[16] Wu JY Shih JY Yang CH Second-line treatments after first-line gefitinib therapy in advanced non-small cell lung cancer. Int J Cancer. 2010; 126: 247-255 19536777 10.1002/ijc.24657

[17] Deng QF Su B Zhao YM Sensitivity of two cell lines with acquired resistance to gefitinib to several chemotherapeutic drugs. Zhonghua Zhong Liu Za Zhi. 2008; 30: 813-816 (in Chinese) 19173824

[18] Becker A Crombag L Heideman DA Retreatment with erlotinib: Regain of TKI sensitivity following a drug holiday for patients with NSCLC who initially responded to EGFR-TKI treatment. Eur J Cancer 2011; 47: 2603-2606 21784628 10.1016/j.ejca.2011.06.046

[19] Jiang SX Yamashita K Yamamoto M EGFR genetic heterogeneity of nonsmall cell lung cancers contributing to acquired gefitinib resistance. Int J Cancer 2008; 123: 2480-2486 18785203 10.1002/ijc.23868

[20] Therasse P Arbuck SG Eisenhauer EA New guidelines to evaluate the response to treatment in solid tumors. European Organization for Research and Treatment of Cancer, National Cancer Institute of the United States, National Cancer Institute of Canada. J Natl Cancer Inst 2000; 92: 205-216 10655437 10.1093/jnci/92.3.205

[21] Maruyama R Wataya H Seto T Treatment after the failure of gefitinib in patients with advanced or recurrent non-small cell lung cancer. Anticancer Res 2009; 29: 4217-4221 19846976

[22] Shoji F Yano T Yoshino I The characteristics and failure pattern of gefitinib responders with postoperative recurrence of pulmonary adenocarcinoma. Eur J Surg Oncol 2008; 34: 89-93 17449217 10.1016/j.ejso.2007.03.005

[23] Riely GJ Kris MG Zhao B Prospective assessment of discontinuation and reinitiation of erlotinib or gefitinib in patients with acquired resistance to erlotinib or gefitinib followed by the addition of everolimus. Clin Cancer Res 2007; 13: 5150-5155 17785570 10.1158/1078-0432.CCR-07-0560

[24] Hata A Katakami N Yoshioka H Erlotinib after gefitinib failure in relapsed non-small cell lung cancer: Clinical benefit with optimal patient selection. Lung Cancer 2011; 74: 268-273 21529987 10.1016/j.lungcan.2011.03.010

[25] Hotta K Kiura K Takigawa N Association between poor performance status and risk for toxicity during erlotinib monotherapy in Japanese patients with non-small cell lung cancer: Okayama Lung Cancer Study Group experience. Lung Cancer 2010; 70: 308-312 20416970 10.1016/j.lungcan.2010.03.008

[26] Asami K Kawahara M Atagi S Duration of prior gefitinib treatment predicts survival potential in patients with lung adenocarcinoma receiving subsequent erlotinib. Lung Cancer 2011; 73: 211-216 21272953 10.1016/j.lungcan.2010.12.014

